# Three Cases of Lactic Acidosis Caused by Biguanides

**DOI:** 10.7759/cureus.31419

**Published:** 2022-11-12

**Authors:** Masaatsu Kuwahara, Hiroko Otagaki, Hideaki Imanaka

**Affiliations:** 1 Department of Emergency Medicine, Takarazuka City Hospital, Takarazuka, JPN

**Keywords:** biguanides, lactic acidosis, diabetes mellitus, case report, acute care medicine

## Abstract

Biguanides may cause lactic acidosis (LA) in elderly patients. We report three cases of LA after the administration of biguanides. Case 1 was an 85-year-old man with no hepatic dysfunction who was discharged, case 2 was a 67-year-old man with no hepatic dysfunction who was discharged, and case 3 was a 77-year-old woman with hepatic dysfunction who died. Therefore, caution should be exercised in administering biguanides to elderly patients with hepatic dysfunction.

## Introduction

Diabetes mellitus (DM) affects 537 million people worldwide [1]. Oral hypoglycemic biguanides are the standard of care for type 2 diabetes. They improve hyperglycemia by decreasing insulin resistance, inhibiting gluconeogenesis in the liver, and promoting glucose uptake by muscle and fat cells [2]. However, metformin (primary biguanide) may cause lactic acidosis (LA) in elderly individuals and patients with hepatic or renal insufficiency.

In the absence of acute overdose, metformin-associated lactic acidosis (MALA) rarely occurs in patients without comorbidities. A systematic review of 347 trials and cohort studies involving 47,846 patient-years revealed no cases of acidosis, with an upper limit of 4.3 cases of LA per 100,000 patient-years [3]. However, in rare MALA cases, the mortality rate is high [4,5]. In a case series of 49 metformin-treated patients, the mortality rate was 45% [5]. Neither blood gas lactate levels nor plasma metformin concentrations can predict mortality, which was closely correlated with underlying comorbidities.

Here, we describe three elderly patients with diabetes with or without hepatic or renal hypofunction who presented to our hospital with marked LA.

## Case presentation

Case 1

Case 1 involved an 85-year-old man weighing 61 kg. Before admission, he was unable to eat for two to three days and ingested only oral medications. He had a history of DM, cerebral infarction, hypertension, and chronic kidney disease and was taking buformin (50 mg × three/day, regular dose).

On arrival, he had a consciousness level on the Japan Coma Scale (JCS) of III-200 [6], blood pressure (BP) of 122/86 mmHg, and blood oxygen saturation (SpO2) level of 100% (oxygen mask at 8 L/min).

Arterial blood gas (ABG) analysis on admission showed considerably high LA: pH of 6.95; partial pressure of carbon dioxide (PaCO2) of 13 mmHg; partial pressure of oxygen (PaO2) of 125 mmHg; bicarbonate (HCO3) of 2.8 mmol/L; base excess (BE) of -28.1 mmol/L; potassium of 7.4 mmol/L; lactate of 17.3 mmol/dL; and plasma glucose (BS) level of 323 mg/dL. Prothrombin time (PT) was normal at 70%. Renal dysfunction was observed: creatinine (Cre) at 6.2 mg/dL.

Immediately after admission to the intensive care unit (ICU), his systolic BP dropped to 50 mmHg and SpO2 was 80% under 10 L/min of oxygen; he was intubated and ventilated. Circulatory agonists (noradrenaline, vasopressin, and adrenaline) were administered to restore his circulation. However, his LA did not improve, and continuous hemodiafiltration (CHDF) was started five hours after ICU admission to remove buformin, buffer with bicarbonate ions, and maintain pH. Lactate levels gradually decreased after five hours of CHDF; after 13 hours, we could reduce the circulatory agonist dose and terminate the CHDF on day three of treatment. He was weaned off the ventilator on day five, discharged from the ICU, and transferred to a rehabilitation hospital on day 22.

Case 2

Case 2 involved a 67-year-old man with a history of heavy drinking. He was brought to the emergency room after a fall in the street caused by unsteadiness when walking. He had a history of DM and hypertension and took metformin (250 mg × two/day, regular dose).

On arrival, he had a consciousness level of JCS 0, BP of 97/49 mmHg, and SpO2 of 94% (room air). ABG analysis showed LA: pH of 7.16; PaCO2 of 27 mmHg; PaO2 of 75 mmHg; HCO3 of 9.4 mmol/L; BE of -17.8 mmol/L; lactate of 15.8 mg/dL; and BS of 163 mg/dL. PT was normal at 134%. The serum Cre level was 1.5 mg/dL, showing mildly impaired renal function.

Excessive alcohol consumption and a metformin prescription led us to suspect LA development. The patient underwent CHDF to remove metformin, buffer with bicarbonate ions, and maintain pH. LA was quickly resolved. The patient was weaned off CHDF on day two and discharged from the hospital on day four.

Case 3

Case 3 was a 77-year-old woman with a history of DM, hypothyroidism, primary biliary cirrhosis, and takotsubo cardiomyopathy. She was taking metformin (500 mg × two/day, regular dose).

The patient arrived at our hospital after experiencing convulsions and had a decreased level of consciousness (JCS: III-300), BP of 120/70 mmHg, and SpO2 of 100% (oxygen mask, 6 L/min) during intravenous infusion of extracellular fluid replacement due to vomiting and diarrhea at a nearby physician's office.

ABG analysis indicated marked LA and hypoglycemia: pH of 7.06; PaCO2 of 36 mmHg; PaO2 of 145 mmHg; HCO3 of 9.8 mmol/L; BE of -20.4 mmol/L; lactate of 18.2 mg/dL; and BS of 6 mg/dL. Liver dysfunction was observed with a PT of 21%. There were no abnormal findings on computed tomography head imaging.

After administering glucose, her level of consciousness improved to approximately JCS II-30. No additional improvement was observed. Bicarbonate administration, CHDF, ventilation, and circulatory agonists were administered. Her condition did not improve and she died on day three (Table 1).

**Table 1:** Summary of patient background and outcome PT: prothrombin time; Cre: creatinine.

	Age	Gender	Biguanide	Liver dysfunction	PT (%)	Renal dysfunction	Cre (mg/dl)	pH	Lactate (mmol/L)	Outcome
Case 1	85	Male	Buformin 150 mg/day	No	70	Yes	6.2	6.95	17.3	Survival
Case 2	67	Male	Metformin 500 mg/day	No	134	Yes	135	7.16	15.8	Survival
Case 3	77	Female	Metformin 1000 mg/day	Yes	21	Yes	0.98	7.06	18.2	Death

## Discussion

We presented three biguanide-associated LA cases, particularly MALA. The mechanism of MALA is complex [3,7]. Among the three patients, two with PT in the normal range at presentation survived, but one patient with low PT died. Thus, we recommend measuring PT when examining patients with MALA.

In a case series of 66 patients with MALA, metformin levels correlated with creatinine and lactate levels, while absolute metformin and lactate levels did not differ between survivors and non-survivors [3].

Metformin levels could not be measured at our hospital. Hence, we cannot speculate on the relationship between metformin levels and mortality. Obtaining serum metformin levels is not the standard of care; timely results are rarely available, and serum concentrations often do not correlate with the severity of poisoning or patient outcomes [3,5]. A risk score considering severe comorbidities in addition to severe acidosis (<7.0) and high lactate levels (>10 mmol/L) may help identify individuals at high risk [8]. Therefore, serum metformin concentrations need not be actively measured. The patient with the highest lactate level reported here died in the hospital.

In chronic metformin use and aggravation of MALA, the most common complaints are gastrointestinal symptoms (nausea, vomiting, and diarrhea) followed by altered mental status and shortness of breath [3]. Of the three patients reported, two presented with gastrointestinal symptoms.

Altered mental status may occur due to acidosis or rare hypoglycemia [9]. One of our patients died as a result of a BS level of 6 mg/dL. As hypoglycemia rarely occurs due to the use of biguanides alone, we assumed that hypoglycemia was caused by other medications (teneligliptin hydrobromide hydrate).

All three patients underwent hemodialysis as recommended in cases of metformin intoxication with elevated lactate levels, marked acidosis, and renal or hepatic insufficiency [10]. We believe that the appropriate use of hemodialysis saved the lives of the two survivors.

## Conclusions

Three elderly patients with biguanide-associated LA were reviewed in this study. Of these, one patient with complicating hepatic dysfunction and extended PT died. Age and/or impaired liver function may be risk factors for high mortality in biguanide-associated LA. Accordingly, hepatic dysfunction should be closely monitored in such cases.
